# Satisfactory spinal anesthesia with a total of 1.5 mg of bupivacaine for transurethral resection of bladder tumor in an elderly patient

**DOI:** 10.1186/s40981-016-0030-0

**Published:** 2016-04-02

**Authors:** Yoshimichi Namba, Michiaki Yamakage, Yoshinori Tanaka

**Affiliations:** 1 Department of Anesthesiology, Sapporo Medical University Hospital, Nishi 16-chome, Minami 1-jo, Chuo-ku, Sapporo, 060-8543 Hokkaido Japan; 2 Department of Urology, Hokkaido Prefectural Esashi Hospital, 484 Fushikido-cho, Esashi-cho, Hiyama-gun, 043-0022 Hokkaido Japan

**Keywords:** Spinal anesthesia, Elderly patient, Dilution and incremental administration of local anesthetic

## Abstract

Spinal anesthesia is popular for endoscopic urological surgery. Many patients undergoing urological surgery are elderly. It is important to limit the dose to reduce any resultant hemodynamic effect. We present a case in which incremental administration of 0.1 % bupivacaine up to 1.5 mg was sufficient to produce satisfactory spinal anesthesia for transurethral resection of bladder tumor (TURBT).

## Background

Spinal anesthesia produces hypotension more often in elderly patients than in younger patients. Limiting the dose (and thus, extent of anesthetic spread) to the necessary dermatomes reduces the likelihood of side effects [1, 2]. We attempted to reduce the necessary amount of bupivacaine by dilution and incremental administration.

## Case presentation

A 95-year-old man (158 cm, 35 kg) was diagnosed with bladder tumor following macrohematuria and scheduled to undergo transurethral resection of bladder tumor (TURBT). His echocardiogram showed aortic (II/IV), tricuspid (II/IV), mitral (mild) and pulmonic (mild) regurgitation. The left ventricular ejection fraction was 54 %. Pulmonary function testing was impossible due to the inability of the patient to cooperate. The family understood the risk of anesthesia and surgery, accepted the anesthesia and surgery.

Four ml of 0.5 % hyperbaric bupivacaine solution was diluted with 16 ml of normal saline to produce an approximately isobaric 0.1 % solution. A T10 dermatomal level of sensory block was targeted to proceed to surgery. The level of sensory denervation was determined by loss of sensation to pin-prick testing. A combined spinal-epidural needle was inserted at the L4–5 interspace and initially 0.5 ml of 0.1 % bupivacaine solution was intrathecally injected through the spinal needle three times resulted in T10 level of spinal anesthesia, then an epidural catheter was inserted. Midazolam 1 mg was administered against anxiety.

An intra-arterial catheter was placed for continuous monitoring of blood pressure. Dopamine was continuously infused at 1.4–7.1 μg/kg/min to maintain an arterial blood pressure around 140/90 mmHg during surgery. Oxygen was supplemented at 3–5 L/min via face mask in the operating room. The operation was finished uneventfully in 1 h. His leg was paralyzed at the end of surgery but could be moved 2 h after the surgery. Both oxygen and dopamine could also be discontinued 2 h after surgery. The postoperative course was uneventful.

## Discussion

Spinal anesthesia is the most frequently used anesthetic technique for endoscopic urological surgery because the signs and symptoms of water intoxication with fluid overload can be recognized earlier. However, many patients undergoing urological surgery are elderly and have coexisting cardiac and pulmonary disease. It is therefore important to limit the distribution of spinal block to reduce adverse hemodynamic and pulmonary effects in such patients [3, 4].

Baydilek et al. [5] compared continuous spinal anesthesia (CSA) with single-dose spinal anesthesia (SDSA) in geriatric patients undergoing transurethral resection of prostate. In their group SDSA, 2.5 ml of 0.5 % levobupivacaine (12.5 mg) was injected through the spinal needle and in their Group CSA, initially 2 ml of 0.25 % levobupivacaine (5 mg) was injected through the intrathecal catheter. If the level of sensory block had not reached T10, an extra 1 ml of 0.25 % levobupivacaine (2.5 mg) was given through the catheter, this was repeated until T10 block level was reached. The mean effective dose in Group CSA was (8.70 ± 1.63) mg. They found that CSA provided better hemodynamic stability, shorter recovery period and equal anesthetic quality. Similar findings to the study of Baydilek et al. were reported by others with spinal anesthesia for orthopedic lower extremity surgery [6–11], but none of them diluted the local anesthetic solution.

Concentration of local anesthetic in cerebrospinal fluid (CSF) is one of the factors affecting uptake of local anesthetic into neural tissue [1, 2]. We diluted local anesthetic solution to attenuate the intensity of neural blockade.

Density of CSF at 37 °C ranges from 0.9998 g/ml to 1.005 g/ml varying with reports [2, 12–16]. Specific gravity of 0.5 % hyperbaric bupivacaine solution at 20 °C is 1.025–1.031 (AstraZeneca Pharmaceutical Company). Density of normal saline at 20 °C is 1.004 g/ml, and that of distilled water at 20 °C is 0.9982 g/ml (Otsuka Pharmaceutical Company). Calculated density of our diluted 0.1 % solution at 20 °C is 1.0078–1.009 mg/ml. Increasing temperature decreases the density of a solution [1, 2]. Our diluted 0.1 % solution would have been warmed up to patient’s CSF temperature and the density would have been less than 1.0078–1.009 mg/ml. In fact, our diluted anesthetic solution worked as an approximately isobaric solution.

One of the most serious complications of spinal anesthesia is cardiac arrest [17–19]. The cardiologist suggested the possibility of cardiac arrest in this case. Unnecessary high level of spinal anesthesia-induced sympathetic blockade should be avoided. The spread of SDSA is reported to be unpredictable [2]. However, our method, incremental administration of diluted local anesthetic solution, made it possible not only to predict but also to control the spread of local anesthetic.

Many articles suggest that the addition of fentanyl or sufentanil can enhance local anesthesia and thereby reduce the dose of local anesthetics with more stable hemodynamics for urological surgery [3, 20, 21] and other surgery [11, 22–26]. Had we added fentanyl to bupivacaine, it might have reduced the dose of bupivacaine and provided more stable hemodynamics and the need for vasopressors.

## Conclusions

The dose of intrathecal bupivacaine to produce a T10 level is reported to be 10–15 mg [1]. This case report demonstrates that when diluted bupivacaine was given in incremental doses of 0.5 mg, a total of 1.5 mg could be sufficient to produce satisfactory spinal anesthesia to proceed to TURBT in an elderly patient. This lower dose can minimize the risk of spinal anesthesia. Prolonged surgery and postoperative pain can be controlled by the combined spinal-epidural anesthesia.

## Consent

Written informed consent was obtained from patient’s daughter.

## Abbreviations

TURBT: Transurethral resection of bladder tumor
CSA: Continuous spinal anesthesia
SDSA: Single-dose spinal anesthesia
CSF: Cerebrospinal fluid
